# Benign and malignant breast lesions identification through the values derived from shear wave elastography: evidence for the meta-analysis

**DOI:** 10.18632/oncotarget.21124

**Published:** 2017-09-21

**Authors:** Yan Xue, Shuxin Yao, Xiaodong Li, Huarong Zhang

**Affiliations:** ^1^ Department of Ultrasonography, Linyi People’s Hospital, Linyi City, Shandong Province 276000, China; ^2^ Department of Ultrasonography, Heze Municipal Hospital, Heze City, Shandong Province 274000, China; ^3^ Department of Radiology, Linyi People’s Hospital, Linyi City, Shandong Province 276000, China

**Keywords:** shear wave elastography, diagnose, breast, meta-analysis

## Abstract

**Objective:**

The analysis was aimed to evaluate the diagnostic accuracy of shear wave elastography (SWE) for malignant breast lesions through a meta-analysis.

**Materials and Methods:**

Related articles were searched in databases of Pubmed, Embase and Cochrane library. Overall sensitivity and specificity were analyzed with DerSimonian and Laird random effects model. Area under curve (AUC) with corresponding 95% confidence interval were also analyzed to evaluate the diagnostic accuracy of SWE. *P* value < 0.05 predicted the significant heterogeneity between study. Sensitivity and publication bias were assessed as well.

**Results:**

According to the inclusion criteria, 25 articles were selected. In the subgroup analysis, diagnostic sensitivity and specificity of SWE in Asian population were 0.84 (0.79–0.88) and 0.87 (0.84–0.90), respectively, while they were 0.92 (0.86–0.96) and 0.89 (0.84–0.92) in Caucasian population. The diagnostic accuracy of SWE was a little higher for Caucasians than for Asians (0.95 vs. 0.92). The diagnostic sensitivity and specificity of virtual touch tissue quantification (VTTQ) were 0.85 (0.77–0.91) and 0.93 (0.88–0.96), respectively. It showed a little higher value in specificity and summary receiver operating curve (sROC) than that of SWE (0.93 vs. 0.87; 0.95 vs. 0.93). In addition, maximum stiffness exhibited higher detection sensitivity than that of mean stiffness (0.91 vs. 0.85).

**Conclusions:**

SWE serves as an accurate diagnostic technology for discriminating malignant and benign breast lesions.

## INTRODUCTION

Breast cancer is one of serious diseases threatening health in women and is also the major cause of death among women [1, 2]. Annually, about 1.38 million new cases and 458, 000 deaths happen worldwide [3]. Moreover, the occurrence rate of this cancer has risen in recent years. Early detection and diagnosis contribute to reducing mortality and improving prognosis. It is urgent to develop efficient detection technology for breast cancer.

Mammographic screening is a valuable tool for early detection of breast cancer [4]. However, the increase in breast tissue density significantly reduces the diagnostic accuracy [5]. Among other imaging methods, gray-scale ultrasonography is a valuable adjunct technique. It shows highly sensitive detection of benign breast lesions from malignant ones [6–8]. The Breast Imaging Reporting and Data System (BI-RADS) along with ultrasonography contribute to understanding the standardized terminology about ultrasonography features, assessments and recommendations [9, 10]. Nevertheless, this technique is subjective and poorly specific [10–12]. Ultrasound elastography emerges as an efficient tool to detect malignant solid lesions through measuring the stiffness. It exhibits 86.5% sensitivity, 89.8% specificity and 88.3% accuracy in discriminating benign and malignant breast lesions [13]. In the ultrasound elastography test, the performance was conducted with freehand compression. The elasticity map largely depends on the the extent of tissue compression and organ’s compressibility limits. Moreover, the differences in skill of the operator may result in distinct results.

Shear wave elastography (SWE), a newly developed technology, could overcome these above mentioned problems. It is performed by remotely inducing mechanical vibrations via acoustic radiation force produced by a focused ultrasound beam. The displacement induced at the focus produces shear wave which delivers information about viscoelastic properties of the tissue, thus generates the quantitative assessment to elasticity values. Until now, there has been many studies investigating the diagnostic role of SWE in discriminating benign and malignant breast lesions, however, no consistent results were obtained.

The meta-analysis was initiated to get more accurate results, which contributes to the early diagnosis of breast cancer and improvement on the treatments.

## MATERIALS AND METHODS

### Article retrieval

The articles were retrieved in Pubmed, Embase and Cochrane databases. The following search terms were used to retrieve articles: “shear wave elastography”, “SWE”, “acoustic radiation force impulse”, “ARFI”, “virtual touch tissue quantification (VTTQ)”, “VTTQ” and “breast”. The references of retrieved articles were carefully checked for potential ones. Only the articles in English were considered.

### Inclusion criteria

The studies were included if they met the following criteria: (1) The study investigated the role of SWE in the diagnosis of malignant and benign breast lesions. (2) Pathological biopsy or cytological (fine-needle aspiration) test was adopted as gold standard. (3) The data of true-positive (TP), false-positive (FP), false-negative (FP) and true-negative (TN) were provided. The study with larger sample size was included for the studies with overlapping data.

### Information extraction

The following information was extracted by two independent authors: name of first author, sample size, number of breast lesions, number of malignant and benign breast lesions, gold standard, SWE parameters, TP, FP, FN and TN. The ambiguity was solved with discussion.

### Statistics

All the analysis was completed in Stata 12.0 (StataCorp LP, College Station, TX, USA) software. Summary sensitivity and specificity were estimated with DerSimonian and Laird random effects model. Besides, summary receiver operating characteristic (sROC) curve was created according to the odds ratios (ORs) weight of sensitivity and specificity. Meanwhile, area under curve (AUC) with corresponding 95% confidence interval (CI) was analyzed to evaluate the diagnostic accuracy of SWE. *P* value was adopted to evaluate the heterogeneity between studies. *P* < 0.05 indicated significant heterogeneity. Deek’s funnel plot was used to assess the publication bias. Subgroup analysis based on ethnicity, technology and SWE parameters (maximum stiffness, mean stiffness and stiffness ratio) were also conducted.

## RESULTS

### Studies selection and characteristics of included studies

**Figure 1:**
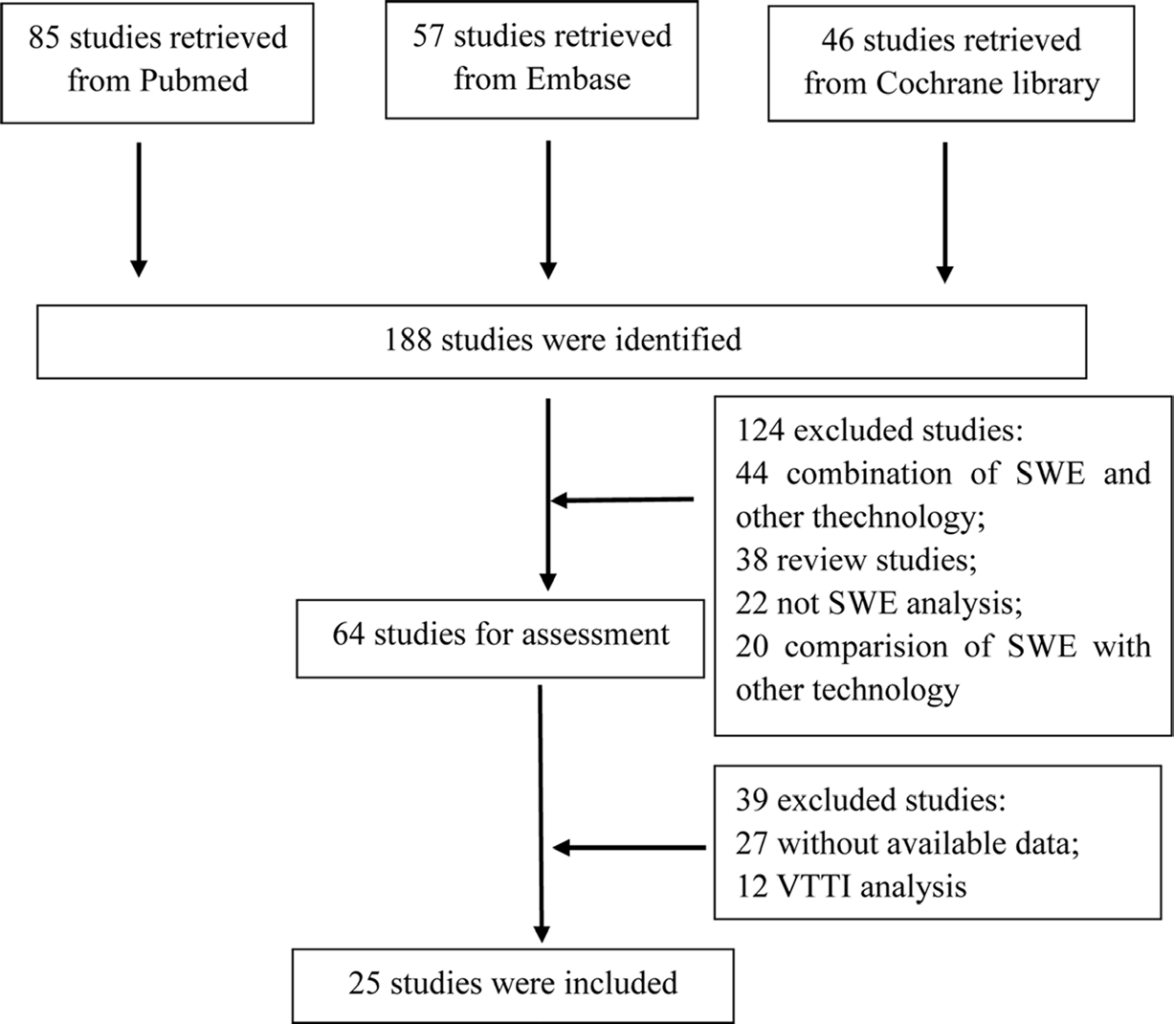
Flow chart of articles selection

The retrieved studies were selected according to inclusion criteria. The selection process was showed in Figure 1. Total of 188 studies were retrieved from databases. Then, 124 studies were excluded for combination of SWE and other technology, review studies, not SWE analysis and comparison with SWE and other technology. Finally, 25 studies [14–38] were included after exclusion of studies for without available data and virtual touch tissue imaging (VTTI) analysis (Table 1). The meta-analysis included 4128 patients and 4546 breast lesions. In the present meta-analysis, 18 articles were for Asian population, while 7 for Caucasian population. 6 articles were based on virtual touch tissue quantification (VTTQ) technology and 19 based on SWE. In the articles of SWE, 13 articles adopted maximum stiffness and 10 adopted mean stiffness.

**Table 1:** Basic characteristics of included studies

Author	Year	Country	Patients, *n*	Lesion, *n*	Benign, *n*	Malignant, *n*	Gold standard	Technology	Parameters
Lo	2015	China	81	88	57	31	Pathology	SWE	-
Xiao	2014	China	93	125	81	44	Pathology	SWE	-
Sobczak	2015	Poland	76	84	43	41	Pathology	SWE	E_mean_
Zhang	2015	China	125	161	106	55	Pathology	SWE	E_max_
Klotz	2014	France	142	167	65	102	Pathology	SWE	E_max_, E_mean_
Au	2014	Canada	112	123	79	44	Pathology	SWE	E_max_, E_mean_, E_ratio_
Yao	2014	China	146	206	163	43	Pathology	VTTQ	SWV
Olgun	2014	Turkey	109	115	83	32	Pathology	SWE	E_max_, E_mean_, E_min_, E_ratio_
Zhou	2014	China	193	193	137	56	Pathology	SWE	E_max_, E_mean_, E_min_
Bai	2012	China	108	143	102	41	Pathology	VTTQ	SWV
Jin	2012	China	95	122	66	56	Pathology	VTTQ	SWV
Meng	2011	China	86	92	65	27	Pathology	VTTQ	-
Tamaki	2013	Japan	180	182	26	156	Pathology	VTTQ	-
Tozaki	2012	Japan	158	161	70	91	Pathology	VTTQ	SWV
Evans	2010	UK	52	53	23	30	Pathology	SWE	E_max_, E_mean_, SD
Chang	2011	Korea	158	182	93	89	Pathology	SWE	E_mean_
Berg	2012	England	939	939	650	289	Pathology	SWE	E_max_
Chang	2013	Korea	129	150	79	71	Pathology	SWE	-
Gweon	2013	Korea	119	133	97	36	Pathology	SWE	SD
Lee^-a^	2013	Korea	139	156	120	36	Pathology	SWE	E_max_
Lee^-b^	2013	Korea	134	144	77	67	Pathology	SWE	E_max_, E_mean_, E_ratio_
Yoon^-a^	2013	Korea	199	222	175	47	Pathology	SWE	E_max_
Yoon^-b^	2013	Korea	236	267	208	59	Pathology	SWE	E_max_, E_mean_, E_ratio_
Youk	2013	Korea	146	163	115	48	Pathology	SWE	E_max_
Evans	2012	UK	173	175	64	111	Pathology	SWE	E_max_, E_mean_

Note: maximum stiffness, E_max_; mean stiffness, E_mean_; minimum stiffness, E_min_; standard deviation, SD; ratio of stiffness of the mass to the background, E_ratio_; shear wave velocity, SWV; VTTQ, virtual touch tissue quantification; SWE, shear wave elastography.

### Summary sensitivity and specificity analysis

**Figure 2:**
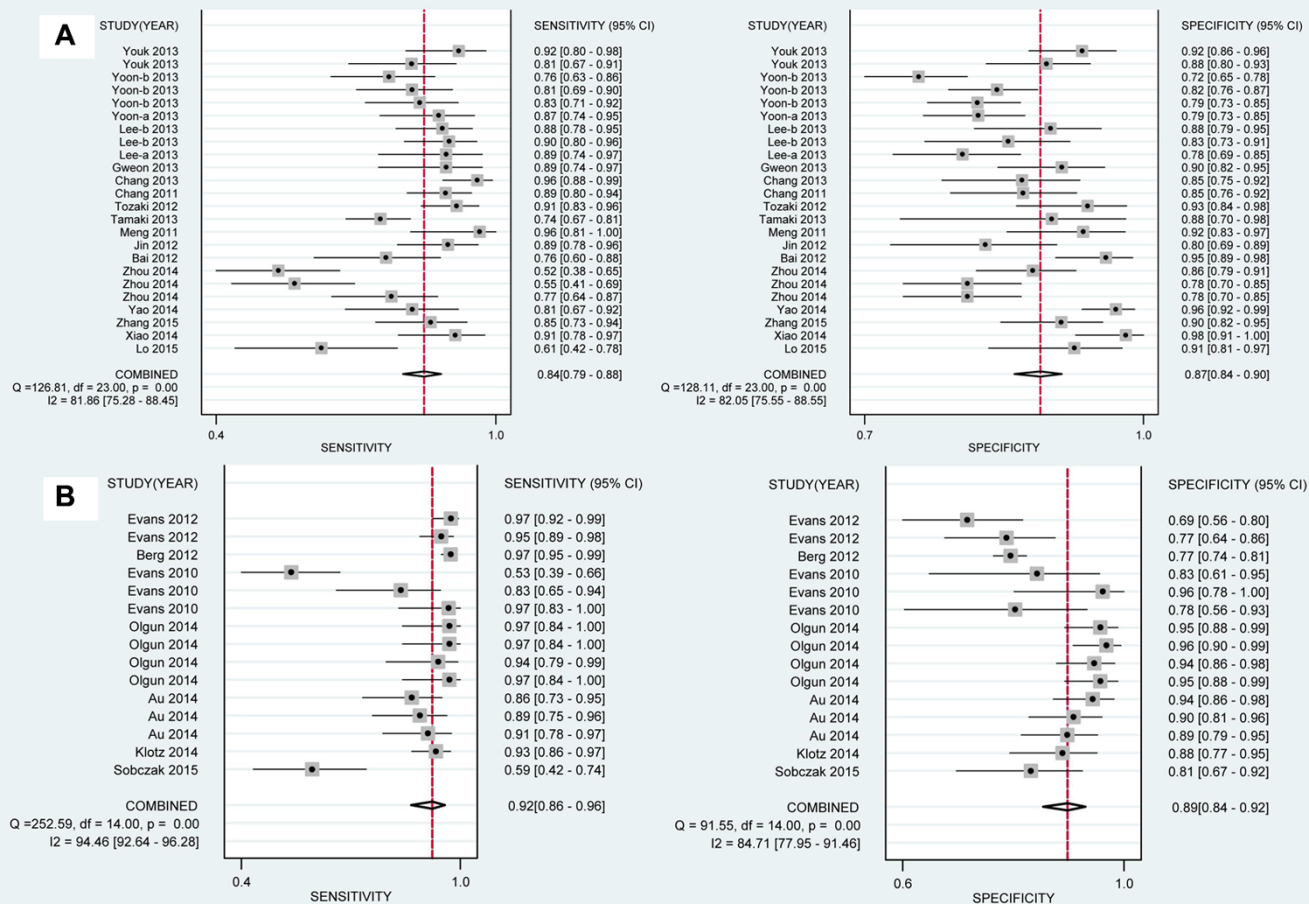
Analysis of the capability for SWE in discriminating breast malignant lesions from benign lesions in subgroup analysis by ethnicity (**A**) Sensitivity [0.84 (0.79–0.88)] and specificity [0.87 (0.84–0.90)] in Asian population. (**B**) Sensitivity [0.92 (0.86–0.96)] and specificity [0.89 (0.84–0.92)] in Caucasian population. SWE shows higher sensitivity and specificity in Caucasians than in Asians.

**Figure 3:**
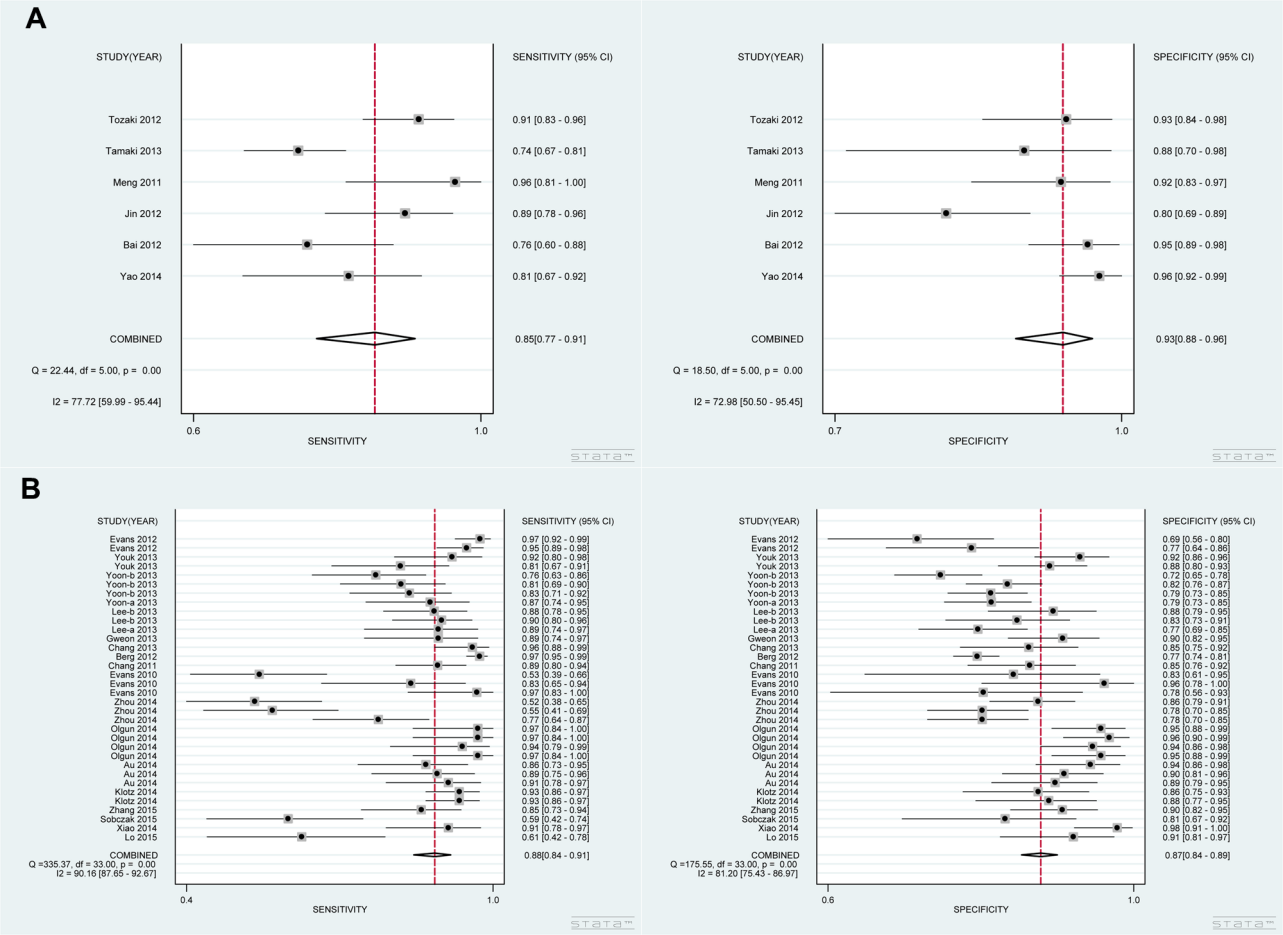
Analysis of the capability for SWE in discriminating breast malignant lesions from benign lesions in subgroup analysis by technology (**A**) Sensitivity [0.85 (0.77–0.91)] and specificity [0.93 (0.88–0.96)] for VTTQ. (**B**) Sensitivity [0.88 (0.84–0.91)] and specificity [0.87 (0.84–0.89)] for SWE. VTTQ shows lower diagnostic sensitivity than SWE, but demonstrates higher specificity than SWE.

**Figure 4:**
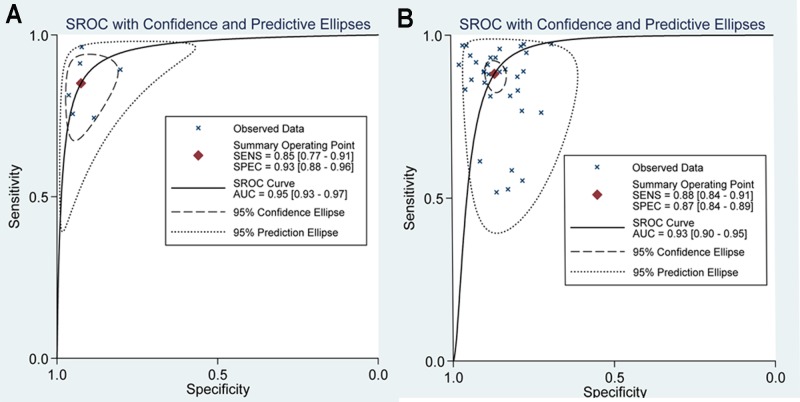
SROC analysis in subgroup analysis by technology (**A**) SROC results for VTTQ. (**B**) SROC results for SWE. The value of AUC is higher for VTTQ [0.95 (0.93–0.97)] than for SWE [0.93 (0.90–0.95)].

The diagnostic sensitivity and specificity of SWE were analyzed, and the results focused on the subgroup analysis based on ethnicity, technology and SWE parameters in Table 2 and Figures 2, 3. In the analysis of ethnicity, the detection sensitivity and specificity of SWE in Asian population were 0.84 (0.79–0.88) and 0.87 (0.84–0.90), the data in Caucasians were 0.92 (0.86–0.96) and 0.89 (0.84–0.92) (Figure 2). So the detection sensitivity and specificity of SWE in Caucasians were higher than that in Asians. According to SROC, AUC was 0.92 (0.90–0.94) in Asians and 0.95 (0.93–0.97) in Caucasians (Figure 4). Therefore, we found that the accuracy rate of diagnosis of SWE in benign and malignant breast lesions identification was also higher in Caucasian than in Asian populations. The subgroup analysis by technology, the results showed the detection sensitivity and specificity of VTTQ were 0.85 (0.77–0.91) and 0.93 (0.88–0.96), meanwhile, the detection sensitivity and specificity of SWE were 0.88 (0.84–0.91) and 0.87 (0.84–0.89) (Figure 3) respectively. We can see the detection sensitivity of SWE was high, but the specificity was low, compared with VTTQ. Based on SROC, AUC of VTTQ was 0.95 (0.93–0.97) and in SWE was 0.93 (0.90–0.95). the accuracy rate of diagnosis of VTTQ was slightly higher than that of SWE, but the difference was not significant. In addition, we investigated the diagnostic role of SWE parameters (maximum stiffness, mean stiffness and stiffness ratio). According to our synthesized data, maximum stiffness exhibited higher detection sensitivity than both stiffness ratio and mean stiffness (0.91 vs. 0.88 vs. 0.85, respectively); while stiffness ratio showed higher specificity than maximum stiffness and mean stiffness (0.88 vs. 0.84 for both comparisons).

**Table 2:** Subgroup analysis of meta-analysis

Subgroup	Sensitivity (95% CI)	P_h_	Specificity (95% CI)	P_h_
Ethnicity				
Asian	0.84 (0.79–0.88)	0.00	0.87 (0.84–0.90)	0.00
Caucasian	0.92 (0.86–0.96)	0.00	0.89 (0.84–0.92)	0.00
Technology				
SWE	0.88 (0.84–0.91)	0.00	0.87 (0.84–0.89)	0.00
VTTQ	0.85 (0.77–0.91)	0.00	0.93 (0.88–0.96)	0.00
SWE Parameters				
E_max_	0.91 (0.87–0.94)	0.00	0.84 (0.80–0.87)	0.00
E_mean_	0.85 (0.71–0.93)	0.00	0.84 (0.79–0.88)	0.01
E_ratio_	0.88 (0.80–0.93)	0.02	0.88 (0.77–0.94)	0.00

Notes: *P*_h_, *P* value for heterogeneity.

### Sensitivity and publication bias analysis

**Figure 5:**
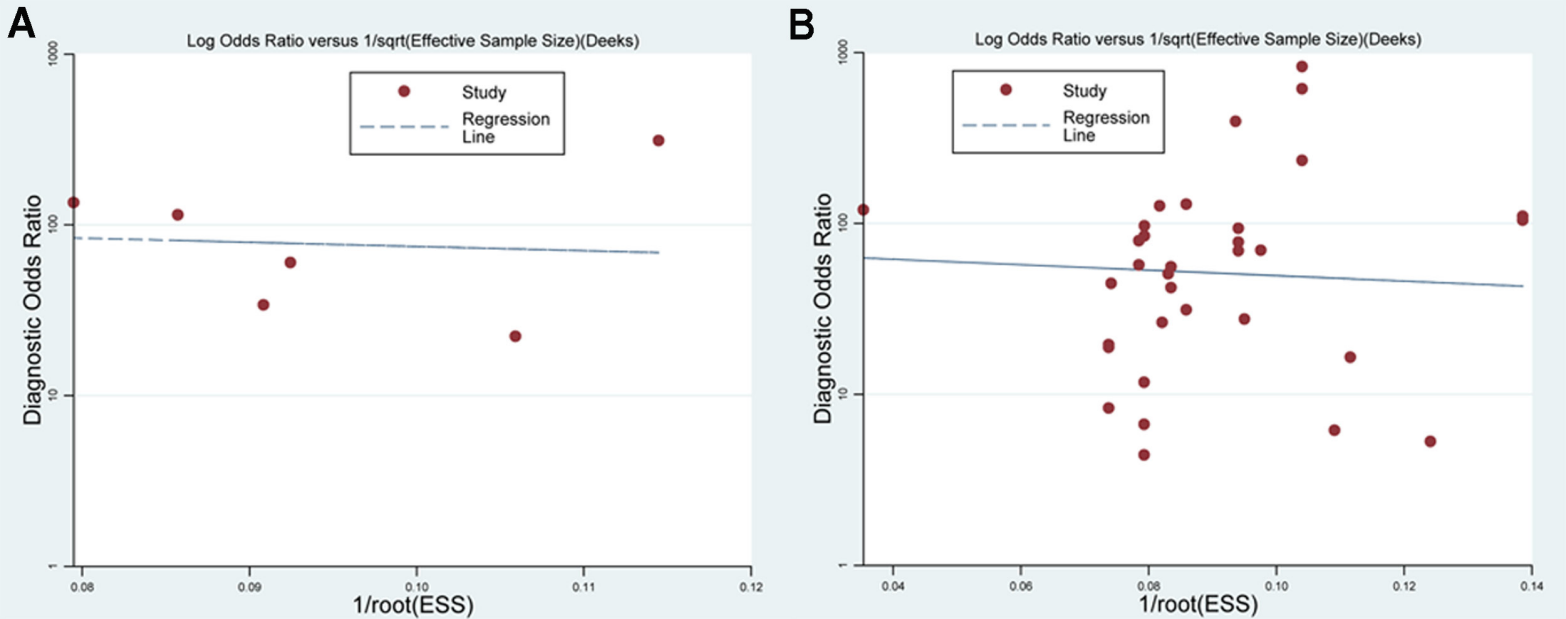
Deek’s funnel plot in subgroup analysis of technology (**A**) Funnel plot for VTTQ (*P* = 0.216). (**B**) Funnel plot of SWE (*P* = 0.080).

Sensitivity analysis was performed by deleting one study each time to observe the changes of results. The analysis indicated that the results were stable. Moreover, no publication bias was found in the meta-analysis (VTTQ: *P* = 0.216; SWE: *P* = 0.08, Figure 5).

## DISCUSSION

SWE is a highly reproducible technology [39]. It determines the propagation velocity of shear waves within the tissues to quantify the stiffness in kPa or m/s [29, 40]. Many tissue elasticity characters can be determined within the region-of-interest (ROI), including maximum (E_max_), mean (E_mean_) and minimum (E_min_) stiffness, standard deviation (SD) and ratio of stiffness of the mass to the background (E_ratio_). Qualitative SWE pattern classification is also reported to show good diagnostic performances [30, 32]. E_max_ and E_mean_ refers to the general stiffness of the mass, while E_ratio_ represent the relative stiffness of the mass to the fat tissue, the elasticity value of which is 3 kPa [28]. SD and pattern classification illustrate the internal heterogeneity of the mass [32], as the malignant masses are almost histologically heterogeneous. The quantitative measurements of SWE have been recognized as more objective information about the breast mass [39, 40].

Among the included studies, the evaluation about SWE in discriminating malignant and benign breast lesions was controversial. In the study of Zhou et al. [22], 193 women with 193 breast lesions were included to analyze the diagnostic performance of SWE in discriminating benign and malignant breast lesions, in which E_max_, E_mean_ and E_min_ were adopted to represent tissue stiffness. However, the diagnostic sensitivity (0.52, 0.55 and 0.77) and specificity (0.86, 0.78 and 0.78) of these three parameters were all low compared with other studies. Meanwhile, Youk et al. [37] reported high detection sensitivity (0.92) and specificity (0.92) of SWE, in which E_max_ represent tissue elasticity. Evans et al. (2012) [38] found that the detection sensitivity of SWE was 0.97 (0.92–0.99), while specificity was only 0.69 (0.56–0.80). On the contrary, Evans et al. (2010) [28] reported 0.53 detection sensitivity and 0.83 detection specificity. The variances in results may lie on the differences in characters of patients, ethnicity or SWE parameters.

Subgroup analysis based on ethnicity, technology and SWE parameters was performed in our analysis. The diagnostic sensitivity, specificity and AUC of SWE in Caucasian population were all higher than in Asian population. As we all know, acoustic radiation force impulse (ARFI) includes VTTI and VTTQ. The result of VTTI is featured by elastographic image, while the result of VTTQ is quantitated by SWV (m/s). Soft tissue shows slow SWV, compared to hard tissue [41]. VTTQ has been used for diagnosis in thyroid, prostate, pancreas, liver and breast [42–46]. In our study, subgroup analysis according to technology (VTTQ and SWE) was conducted. VTTQ showed higher detection specificity and accuracy than SWE, but its sensitivity was lower than SWE. So, the result is usually unsatisfactory using single technique for breast lesions identification and combination of SWE and VTTQ may be a good technique. In terms of SWE parameters, E_max_ showed higher diagnostic sensitivity than E_mean_ and E_ratio_, and E_ratio_ possessed higher specificity than the other two parameters.

Due to the different study population, sample size, exploration factors or examinee-level errors of measurement, the diagnosis results presented in every eligible study were inconsistent. Thus the meta-analysis was the common method to solve the controversy as much as possible. This meta-analysis was based on 4128 patients and 4546 breast lesions. The results were reliable and stable. However, some defects must be pointed out. The number of articles based on Caucasian population was much less than that of Asian population. The accuracy of results on Caucasian population might be affected. In our analysis, the performance of SWE seemed to be better in Caucasians than in Asians, especially for sensitivity, and this phenomenon need to be explored in future special researches, considering the interested topic and space limitation in the present meta-analysis. In previous study, the diagnosis technique of breast lesions distinguish was also different by ethnicity. The diagnostic accuracy of breast lesions based on ultrasound elastography (UE) was 88.4% in Japanese [47] and the percentage was 80.6% in Chinese. Therefore, the diagnosis accuracy difference of the same technique by different races have to be allowed to exist because of variant genetic background and relative environmental factors. In addition, there existed significant heterogeneity between studies. The heterogeneity might result from multiple internal and external factors, such as different threshold values adopted in individual studies, uneven patients’ number, various basic features of patients and diverse test conditions. However, limited to confined information from our included studies, further subgroup analyses based on age and other potentially relevant factors were not performed, so possible sources for the heterogeneity were not identified in this meta-analysis.

Our meta-analysis demonstrates that SWE is an accurate and reliable diagnostic tool in discriminating malignant and benign breast lesions. The outcome is significant in clinic, which contributes to the early diagnostic of breast cancer.
